# A general model of conversational dynamics and an example application in serious illness communication

**DOI:** 10.1371/journal.pone.0253124

**Published:** 2021-07-01

**Authors:** Laurence A. Clarfeld, Robert Gramling, Donna M. Rizzo, Margaret J. Eppstein

**Affiliations:** 1 Department of Computer Science, University of Vermont, Burlington, VT, United States of America; 2 Department of Family Medicine, University of Vermont, Burlington, VT, United States of America; 3 Department of Civil and Environmental Engineering, University of Vermont, Burlington, VT, United States of America; 4 Vermont Complex Systems Center, University of Vermont, Burlington, VT, United States of America; Hong Kong Polytechnic University, HONG KONG

## Abstract

Conversation has been a primary means for the exchange of information since ancient times. Understanding patterns of information flow in conversations is a critical step in assessing and improving communication quality. In this paper, we describe **CO**nversational **DY**namics **M**odel (CODYM) analysis, a novel approach for studying patterns of information flow in conversations. CODYMs are Markov Models that capture sequential dependencies in the lengths of speaker turns. The proposed method is automated and scalable, and preserves the privacy of the conversational participants. The primary function of CODYM analysis is to quantify and visualize patterns of information flow, concisely summarized over sequential turns from one or more conversations. Our approach is general and complements existing methods, providing a new tool for use in the analysis of any type of conversation. As an important first application, we demonstrate the model on transcribed conversations between palliative care clinicians and seriously ill patients. These conversations are dynamic and complex, taking place amidst heavy emotions, and include difficult topics such as end-of-life preferences and patient values. We use CODYMs to identify normative patterns of information flow in serious illness conversations, show how these normative patterns change over the course of the conversations, and show how they differ in conversations where the patient does or doesn’t audibly express anger or fear. Potential applications of CODYMs range from assessment and training of effective healthcare communication to comparing conversational dynamics across languages, cultures, and contexts with the prospect of identifying universal similarities and unique “fingerprints” of information flow.

## Introduction

Conversation is a fundamental form of human communication. Conversations are highly complex phenomena [1], but use simple rules to maintain discourse [2]. Humans have an innate ability to learn spoken language from infancy, yet despite the importance of conversations in our daily lives, achieving effective communication through conversations can be difficult [3, 4]. Not only is the informational content of conversation important for effective communication, but also the manner in which the information is exchanged. For example, in serious illness conversations, it is important for the clinician to convey empathy and to help establish a sense of trust and understanding with the patient. Developing a better understanding of information flow in different conversational contexts, such as during moments of strong emotional content and connection, can help guide efforts to improve conversation quality.

Conversation analysis (CA) became established as a discipline of study beginning in the late 1970’s with the seminal work of Harvey Sacks and others, such as in their formative 1978 paper [2], in which they present a framework for the process of conversation. Sacks described conversation as being highly structured around turn-taking, with participants being able to fluidly transition between turns while minimizing long gaps and interruptions. This fundamental property of conversation has subsequently been observed and measured across languages and cultures, revealing itself to be a universal trait [5]. Sacks theorized that this discourse is maintained by a set of rules, or norms, that are followed by participants and govern when the speaking floor is relinquished and which participant may speak next. Perhaps the most important aspect of his framework is the heavy focus on the sequential nature of conversation and the dependence of each speaker turn on the turns that came before it [2].

The traditional conversation analytic approach to understanding sequence is to use meticulous transcriptions of recorded conversations in order to study a pre-specified conversational phenomenon and understand its normative patterns [6, 7]. For example, in one early, influential study, Schegloff examined the opening sequence of turns in 500 telephone calls and attempted to explain the patterns he observed [8]. This approach has been widely adopted and applied in diverse contexts, resulting in a vast body of work comprising thousands of research papers. While the value and importance of this inherently qualitative approach remains relevant today [6, 7, 9], quantitative methods have gained increasing popularity in CA (e.g., [10, 11]). In particular, Markov Models (MMs) inherently model sequential events [12], and so have been widely applied in CA.

In a MM, the likelihood of a given event occurring is determined by the current state of the system, and when an event does occur it causes the system to transition to a new state [12]. The “order” of a MM defines the number of previous events that are recorded in each state (i.e., the length of the “memory” in the model). In most CA applications of MMs, state transitions are defined to take place between some constant, fixed intervals of time. Examples of these include 1^st^-order MMs that were used to classify dialog scenarios in conversations based on speech/silence states [13], and for identifying conversational structure within non-verbal states, such as gaze patterns in four-person conversations [14], and 2^nd^-order MMs used to study the effects of conversational speech/silence patterns on communication systems [15]. In contrast, sequences of speaker order in four-person conversations were used to predict who the next speaker would be, using MMs up to order 5 [16]; 2^nd^-order MMs proved significantly better than 1^st^-order MMs for this task, but little further improvement was gained by moving to higher order models on this data set, and a simple context-sensitive model based on speaker roles was shown to out-perform the MMs.

While the examples above use MMs as a tool for making predictions or classifications regarding conversations, another approach to understanding the structure of information flow involves classifying units of conversation by their functional roles (e.g., [17, 18]). Once these functional roles have been defined, 1^st^-order Markov models have been used to understand the sequence of these functions in conversation [19–21]. Influence modeling is yet another Markov-based method, where individual Markov chains for each speaker are coupled together to understand how speakers interact, including understanding which speakers are most influential [22–24] and the functional role of each speaker [25].

Visualizing data to aid interpretation has also grown in popularity [26], and a number of methods have been proposed for visualizing conversational dynamics [27–29]. Effective visualizations must transform complex, possibly dynamic, information into semantically interpretable images, allowing expected patterns to be recognized and unexpected patterns to be discovered [30]. One popular tool for visualizing conversational dynamics is Discursis [29], which uses conceptual recurrence plots for unsupervised identification and visualization of shared content between speaker turns in the analysis of conversational discourse. This method has been used in a variety of contexts, including the study of healthcare conversations [31–36]. Discursis visualizations portray the lengths of sequential speaker turns within an individual conversation, along with the amount of overlap in content in these turns. Thus, the size and complexity of Discursis visualizations vary depending on the number and content overlap of the turns being visualized, and access to conversational content is necessary to identify shared topics and create these detailed visualizations of all or part of individual conversations.

We expect that the amount of information conveyed during a given turn will be influenced, in various context-dependent ways, by the amount of information conveyed in previous speaker turns. For example, if one person is “holding the floor” [6] we may expect to see alternating sequences of long turns by the “talker” and short “continuer” turns by the “listener” [37–39]. We expect that conversational openings and closings may consist of a series of short turns; the importance of ritualized openings and closings was explored by some of the early pioneers of conversation analysis [8, 40] and has been the subject of much attention since then [41–45]. Alternatively, we expect that periods of bilateral sharing may consist of a series of long turns. Looking at overall patterns of turn sequence dependencies may provide important insights into the nature of different types of conversations or turns taken within different contexts.

In this paper, we describe the **CO**nversational **DY**namics **M**odel (CODYM), a novel MM approach for analyzing and visualizing high-level patterns of information flow across sequences of turns in one or many conversations, using the length of a speaker turn as a simple proxy for the capacity of information conveyed in the turn. The proposed method is scalable to large conversational corpora and conceptually can be fully automated in real time, with no need to store or transcribe conversations. Since the construction of CODYMs requires only sequences of turn lengths, rather than knowledge of specific conversational content of turns, the privacy of the conversational participants is completely preserved. In addition to word content, there are many other important verbal and non-verbal conversational features that simple turn-length-based CODYMs do not directly incorporate, such as interruptions, overlapping speech, conversational pauses, accent, intonation, gestures, facial expressions and eye contact, all of which play some role in conversational discourse. While additional features *could* theoretically be directly incorporated into CODYM states, the state space of the resulting models would be larger, less well populated, and more difficult to interpret. CODYMs can, however, be contextualized to study information flow patterns surrounding specific topics or contextual events, when such are known. As an example of contextualization, in this paper we show how information flow patterns differ in conversations where distressing emotions are or aren’t audibly detectable.

CODYM analysis is thus general and complementary to existing methods, providing a new tool for use in conversation analysis. In contrast to previous applications of MMs to conversation analysis, CODYMs are the first to use discretized lengths of speaker turns as the fundamental unit of state, and the primary function of CODYM analysis is to quantitatively summarize and visualize high-level information flow patterns throughout one or more conversation(s), rather than to make predictions or classifications. In contrast to Discursis, which provides a more detailed low-level visualization of turn lengths and shared content between turns within individual conversations, CODYMs provide succinct, high-level, abstract, uniformly-sized “fingerprints” of information flow patterns in one or more conversations, thus facilitating comparisons between different corpora and/or different contexts.

We demonstrate CODYMs with an application in serious illness conversation analysis. In healthcare communication, and especially in serious illness communication, the quality of doctor-patient conversations can have profound tangible impacts [46–48]. Promoting high-quality communication in serious illness healthcare is considered a national priority [49, 50], and there is an increasing recognition that automated methods for analyzing clinical conversations, such as turn-taking analysis, could provide useful feedback and insights for improving communication between clinicians and patients [48, 51]. Thus, as an important first application, we apply CODYM analysis to a corpus of transcribed conversations between palliative care clinicians and seriously ill patients, recorded as part of the Palliative Care Communication Research Initiative (PCCRI) [52]. These conversations are dynamic, complex phenomena that take place amidst heavy emotions such as anger and fear [53]. They include difficult topics such as end-of-life preferences and values, all while patients endure suffering from the symptoms and distressing uncertainties of their illness.

In this initial CODYM application, we seek to answer the following questions. Are there normative information flow patterns in serious illness conversations? If so, how do these differ between patients and clinicians, and differ from what would be expected if there were no sequential dependencies in turn lengths? In what ways do those patterns change during the course of a conversation? How does the expression of distressing emotions such as anger or fear impact patterns of information flow? We show that CODYM analysis provides a quantitative approach, with an intuitive interpretation, that helps to answer these questions.

The remaining sections of this manuscript are organized as follows. We first describe the methods involved in CODYM analysis, and the PCCRI corpus of palliative care conversations. We then present results of applying CODYM analysis to the PCCRI corpus to answer the aforementioned questions, followed by a discussion of the significance of our findings. Finally, we conclude with a discussion of various other ways we envision CODYMs could be used to assess, and potentially improve, conversational dynamics in healthcare settings, without compromising patient privacy. These include examining how telehealth consultations may alter communication patterns, assessment of mock consultations for clinicians-in-training, comparing patterns of information flow between different fields of medicine, and studying how communication patterns may vary before and after “connectional silences” [54] in serious illness conversations.

## Methods

### Conversational dynamics model

We use the length of a speaker turn as a simple proxy for the capacity of information that the turn can convey. We define a **CO**nversational **DY**namics **M**odel (CODYM) to be a Markov Model (MM) where each event is a speaker turn of a given discretized length and states consist of some predefined number (defined by the order of the model) of the immediately preceding events. CODYMs thus model the sequential patterns in turn lengths. If working from transcripts, as we do here, turn lengths are based on the number of words in a turn.

Any MM requires a discrete state-space, so turn lengths in a CODYM are discretized into a finite number of bins. Here, we elect to binarize turns lengths around the median turn length. In the PCCRI conversations analyzed here, turn lengths follow a heavy-tailed distribution, with a median turn length of 7 words (S1 Fig). We thus define short (S) turns as those with 1–7 words and long (L) turns as those with 8 or more words. Using the median turn length as the maximum length of short turns (a) minimized the disparity between the number of short *vs*. long turns, and (b) maximized the Shannon entropy (a measure of information content) for the distribution of states in a 3^rd^-order CODYM (S2 Fig). In preliminary experimentation with the PCCRI corpus, we found that using ternary bins (Short/Medium/Long) was problematic because it both (a) created sample size issues by reducing the number of turns associated with each transition, and (b) resulted in more complex models that were more difficult to interpret. Ultimately, the most appropriate discretization of turn lengths depends on the nature of the data being analyzed and the questions being asked.

**Fig 1:**
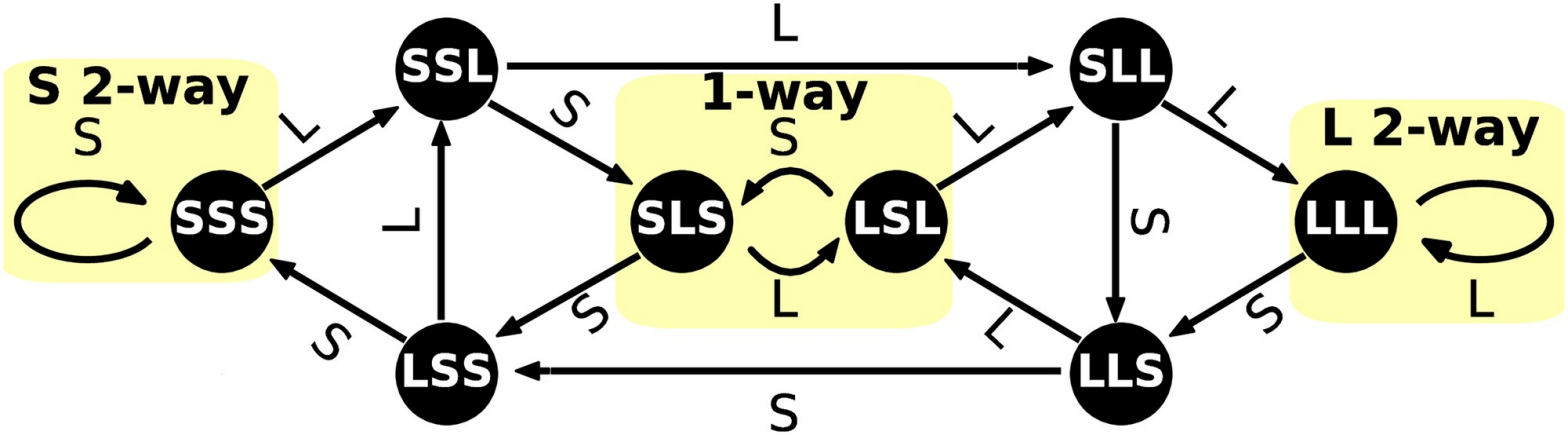
Network representation of a CODYM. Network depiction of a 3^rd^-order CODYM, where turn lengths are binarized as short (S) or long (L). Nodes (black circles) represent states that are defined by the lengths of the 3 previous turns, respectively; edges (arrows) represent transitions between states and are labeled with the length of the turn on that transition. The areas highlighted in yellow represent important sub-networks we refer to as short two-way information exchanges (labeled “S 2-way”), one-way information exchanges (labeled “1-way”), and long two-way information exchanges (labeled “L 2-way”).

One must specify the order of the MM to be used in a CODYM. Note that an *N*^th^-order CODYM of binarized turn lengths has 2^*N*^ states and 2^*N*+1^ transitions. For example, a 3^rd^-order CODYM has 8 states and 16 transitions, as illustrated in Fig 1, where states are represented as nodes and transitions are represented as directed edges in a network.

In this work, we use 3^rd^-order CODYMs for analyzing normative patterns in serious illness conversations, and for examining how these patterns change both temporally and when certain distressing emotions are expressed. We selected 3^rd^-order CODYMs for this because: (a) for dyadic conversations, such as between patients and clinicians, having memory of 3 previous turns can represent a complete back-and-forth exchange between the 2 sides; (b) higher-order models become difficult to interpret; and (c) large state spaces result in fewer observations of each state/transition, potentially resulting in small sample sizes that may preclude accurate characterization of normative patterns (e.g., the median numbers of occurrences of the different states for each conversation analyzed here are 44, 22, 10, and 5, for CODYMs of orders 2–5, respectively).

Different sub-networks of a CODYM can be interpreted as distinct regimes of information flow (highlighted in yellow in Fig 1). In this work, we refer to the center loop ($\text{SLS}\overset{L}{\to }\text{LSL}\overset{S}{\to }\text{SLS}$) as “one-way information exchange”, because alternation between S and L turns in a dyadic conversation implies that one party is conveying most of the information. We refer to the leftmost self-loop ($\text{SSS}\overset{S}{\to }\text{SSS}$) as “short two-way information exchange”. Conversely, we refer to the rightmost self-loop ($\text{LLL}\overset{L}{\to }\text{LLL}$) as “long two-way information exchange”.

### Observing patterns of information flow

In most MMs, the weights on all outgoing edges of each given node sum to 1.0, where each edge weight represents the probability that the node is left *via* that edge. This is appropriate when MMs are used as generative models or to make predictions of future states. However, with CODYMs our primary intent is to study patterns of information flow through all states and transitions in an existing corpus of dialog, not to generate simulated sequences of short and long speaker turns or to predict subsequent turn lengths. Thus, we “populate” a CODYM by computing observed frequencies of each state/transition across a specified set of speaker turns. This set may comprise all turns in an entire corpus of conversations, all turns for a given speaker, all turns within individual conversations, or some other subset of turns that satisfy some pre-specified condition, depending on the question being addressed. In a populated CODYM, the weights on nodes and edges represent their respective percentage frequencies of occurrence over all turns being analyzed. Consequently, the sum of all edge weights (transition frequencies) in a populated CODYM is 100% and the sum of all node weights (state frequencies) is also 100%.

When different CODYMs are populated separately for each of a number of conversations, they can be visualized as a single CODYM populated with the mean weights for each of the states and transitions. A CODYM of mean frequencies can be interpreted as a representation of the overall “normative” pattern of information flow in the set of conversations under study, assuming the distributions of frequencies are approximately uni-modal for each state and transition. We elect to create the normative CODYMs with the means (rather than medians) of transition and state values, since this preserves the more intuitive properties that (a) the sum of all edge weights in each CODYM of means remains 100%, (b) the sum of all node weights in the CODYM of means remains 100%, and (c) for each node in a CODYM of means, the sum of weights of incoming edges still equals the sum of the weights of outgoing edges.

In this study, we initially create separate CODYMs for patient turns and for clinician turns, within each of the serious illness conversations being analyzed. We use Mann Whitney U tests to determine whether the corresponding state and transition frequency distributions differ significantly between patients and clinicians. We visualize the normative patterns for patients and clinicians by averaging the observed frequencies in these populated CODYMs over all of the conversations.

The null hypothesis is that turn lengths are independent between turns. We test this hypothesis for the normative CODYMs of both patients and clinicians, by comparing to null models of mean frequencies. The odds of each state or transition occurring are not equal in a null model when there are differences in the number of words spoken by different speakers. For example, in this particular dataset, only 41% of patient turns are long whereas 51% of clinician turns are long. Thus, to create appropriate null models, we first randomly shuffle the lengths of all patient turns and the lengths of all clinician turns, while maintaining the original sequential order of patient and clinician turns, on a conversation-by-conversation basis. This preserves the exact overall distributions of patient and clinician turn lengths but deliberately breaks any non-random sequential dependencies in turn lengths. Null CODYMS are then created, for both patient turns and clinician turns, using each of the conversations’ randomized sequences of turn lengths. State and transition frequencies are subsequently averaged over the null CODYMs for all conversations. We repeat this process 1000 times, randomly reordering the turn lengths each time, to derive empirical probability distributions for the means of state/transition frequencies in null models. (In preliminary experimentation we observed that the probability distributions of mean frequencies were stable and normally distributed when created with 1000 simulations). We compare observed means to these empirical distributions of means in the null models to estimate the probabilities that the observed means could have arisen by chance, given the observed frequencies of long and short turns. For example, if an observed mean state/transition frequency is outside of the empirically derived 95% confidence interval for the mean of the same state/transition in these null distributions, the difference is considered to be statistically significant at the *P* <0.05 level; if observed means are completely outside of the range of the null distributions, they are considered to be statistically significant at the *P* <0.001 level.

Normative patterns are illustrated directly using mean observed transition frequencies (%*Observed*). In the normative visualizations, all state weights sum to 100%, all transition weights sum to 100%, and the color bars always represent positive percentages. The thickness and color of transition arrows indicate the magnitude of the corresponding transition frequencies, and nodes are sized according to the frequency of their respective states.

We visually compare two different information flow patterns using difference CODYMs, by subtracting the corresponding state and transition percentage frequencies of one CODYM (the subtrahend) from another (the minuend), to compute the difference (Δfrequency). In the difference visualizations, transition weights sum to 0%, negative values indicate the degree to which the frequency is smaller in the minuend, and positive values indicate the degree to which frequencies are larger in the minuend. The thickness of transition arrows, and diameters of nodes, are sized in proportion to the magnitude of the corresponding differences (|Δfrequency|). The color of nodes and arrows indicates both magnitude and direction of the differences between corresponding states and transitions, respectively. State and transition frequencies that are statistically significantly different between the minuend and the subtrahend at the *P* <0.05 level are indicated by outlining states in black solid (*vs*. dashed) circles and drawing transition arrows with solid (*vs*. dashed) lines.

### Temporal changes in normative patterns

To assess how normative patterns in information flow may change over the course of a conversation, we divide the turns of each conversation in the corpus into sequential deciles of words (ten bins of narrative time, as in [55]), stratified by patient and clinician turns. Note that different conversations have different numbers of turns, so the number of turns per bin varies by conversation. The conversations analyzed here average only about 12 patient turns and 12 clinician turns per decile. Since 12 turns are inadequate to robustly determine frequencies on 16 transitions, we pool the data by summing the number of patient and clinician turns per decile over all conversations. We then compute the frequencies of the pooled turns on each of the 16 transitions in 3^rd^-order CODYMs, one per decile.

### Contextualization of CODYMs by expressions of distressing emotions

A CODYM is based exclusively on turn lengths, so is thus independent of what is actually said or expressed during those turns. However, a CODYM can be used to examine the information flow patterns involving different words, topics, expressions of emotion, or more generally, “contextual events”.

Here, we use CODYMs to compare patterns of information flow over conversations with or without any audible patient expressions of anger or fear. We provide visualizations of the normative CODYM pattern of patient turns in conversations where the patient expressed any fear or anger and of the difference CODYM for patients turns in conversations with expressions of anger or fear minus those without. We use Mann Whitney U tests to determine whether the corresponding state and transition frequency distributions of patient turns differ significantly between conversations with/without any fear or anger.

### The PCCRI corpus

The Palliative Care Communication Research Initiative (PCCRI) is a multisite observational cohort study conducted between January 2014 and May 2016 [52]. The study took place at two large U.S. academic medical centers, one in the Northeast and one in the West. Any English-speaking patients who were hospitalized and referred for inpatient palliative care consultation were eligible for this study, provided they were diagnosed with a metastatic nonhematologic cancer, did not have a documented exclusively comfort-oriented plan of care at the time of referral, were age 21 or over, and were able to consent for research either directly or via health care proxy (if lacking capacity as determined by the clinical team). All members of the interprofessional Palliative Care Inpatient Consult teams at both sites were eligible to participate.

A total of 240 hospitalized patients with advanced cancer at the time of referral for inpatient palliative care consultation were enrolled in the study. Four withdrew, three died, and two were discharged before completing the palliative care consultation. Each consultation comprised one to three conversations between the patient, and potentially family members and/or close friends of the patient, and the palliative care team. More than one conversation occurred with the same patient when the initial conversation was only a preliminary assessment or when a conversation was interrupted prematurely (e.g., a patient was taken for x-rays) [52].

All conversations that were part of a palliative care consultation were audio recorded. With prior informed consent from all study participants, digital recorders were placed in unobtrusive locations in the rooms where the conversations took place (e.g., on a tray table next to a patient’s bed); research assistants retrieved the recorders at the end of the visit by the palliative care team. All audio recordings were later transcribed verbatim and prepared in a standard format to facilitate natural language processing. The speaker during each transcribed turn was tagged as either from the patient side of the conversation (which could include family members and/or close friends who were present in support of the patient) or a clinician, except in rare occasions (<1% of turns) when transcribers could not determine whether the speaker was from the patient side or the clinician side. All speaker turns in which the patient was audibly perceived to be expressing anger or fear were subsequently labeled in the PCCRI transcripts, using well-established and reliable human coding methods [56, 57]. Anger was defined to include expressions of either frustration or anger. Fear was defined to be inclusive of words and sounds indicating worry, anxiety, fear or terror. Turns that included both sentiments were coded as such. Ambiguous words or sounds that *might* indicate underlying emotion, or referred to emotions felt in the past, were not included in our analysis.

In total, 360 conversations were recorded and transcribed for 231 unique patients. Of these, five transcripts were excluded from this study because either a high proportion of speaker turns were inaudible rendering the transcripts very incomplete, or because the conversations were too short (less than 20 speaker turns long) to perform meaningful analyses. Here, we analyze the subset of 117 conversations for which there was exactly one patient-side participant (i.e., no family members or friends participated in the conversation) (see S1 Dataset). These 117 conversations contained 354,422 total words in 27,597 total speaker turns (14,880 short turns and 12,717 long turns), with a median of 175 turns per conversation (S1 Fig). The median length of short turns was 2 words and the median length of long turns was 16 words.

Out of the 117 conversations, 55 (47%) had at least one instance of anger or fear, creating a relatively balanced data set for comparing conversations with anger or fear to those without. In 13 conversations (11.1%) both of these different types of distressing emotion were present. Conversations that had at least one patient turn with a distressing emotion typically (72.4% of the time) had more than one, with a heavy-tailed distribution indicating that a small number of conversations had very high instances of emotion. It was also common to have multiple consecutive patient turns where emotion was expressed. Consequently, the previous turns, used in defining the state prior to a given transition where emotion was present, also often included expressed emotions. This should be considered when interpreting the results herein.

## Results

### Normative patterns of information flow in serious illness conversations

CODYMs reveal normative patterns (“fingerprints”) of information flow in serious illness conversations. The existence of such normative patterns is supported by the approximately uni-model distributions observed for states and transitions in 3^rd^-order CODYMs, for both patients and clinicians, indicating a prevailing pattern across all conversations in the corpus see S3–S5 Figs for the distributions, with means and medians shown, for all state and transition values in each of the 117 PCCRI conversations analyzed).

**Fig 2:**
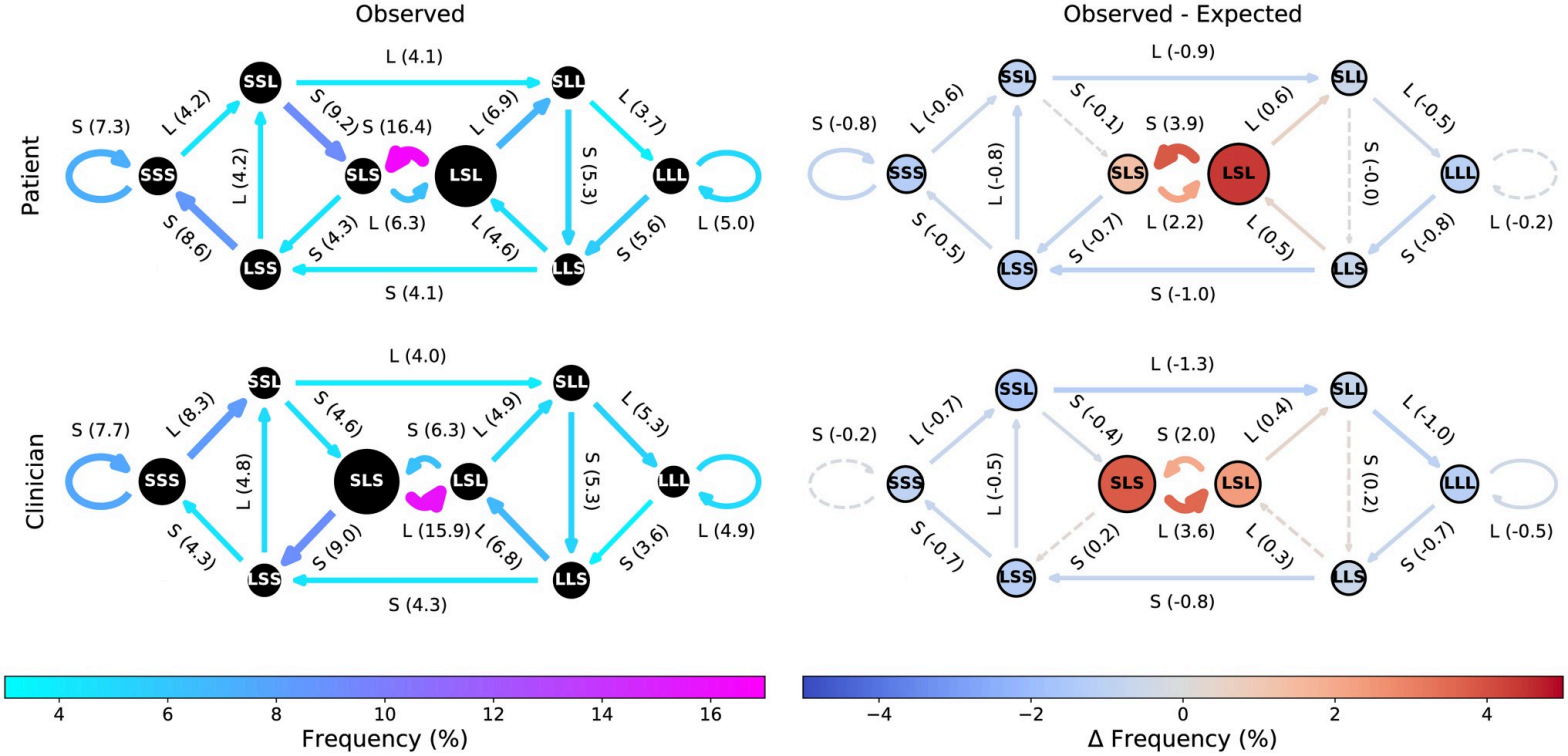
Normative patterns of information flow in the PCCRI corpus. (Left column) CODYMs of normative patterns of information flow for patient turns (top) and clinician turns (bottom), averaged over the 117 PCCRI conversations analyzed (%*Observed*). (Right column) Difference CODYMS, where ΔFrequency = (%*Observed*) − (%*Expected*), for both patient turns (top) and clinician turns (bottom). In the difference CODYMs, state and transition frequencies where observed values are statistically significantly different from expected values (*P* <0.05, by empirically determined 95% confidence intervals for the null models) are indicated by outlining states in black solid (*vs*. dashed) circles and drawing transition arrows with solid (*vs*. dashed) lines.

All states, and a majority of the transitions, of the normative CODYMs for patients and clinicians (Fig 2, left column) differ significantly (*P* <0.05) from the expected values derived from the corresponding null models (Fig 2, right column; the individual *P* values are shown in S1 and S2 Tables). In particular, states SLS and LSL and the transitions between these two states (shown in warm colors), occur much more frequently in the observed data than expected by chance, in both patients and clinicians. This indicates that these serious illness conversations include more one-way information exchanges than would be expected by chance, and that sometimes it is the clinician imparting more information and sometimes it is the patient.

**Fig 3:**
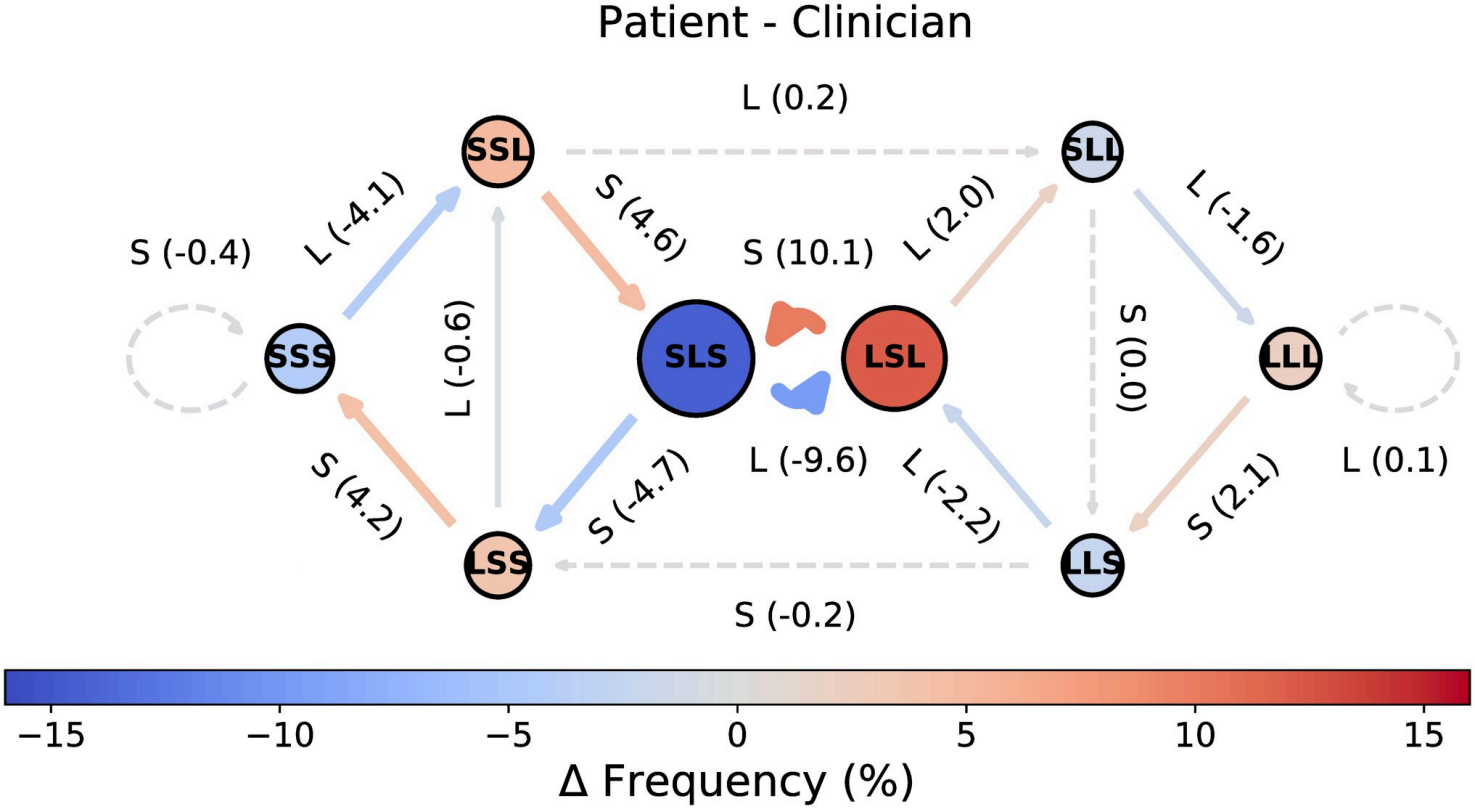
Differences in information flow between patient and clinician turns in the PCCRI corpus. Difference CODYM, where ΔFrequency = (%*Observed* for Patient) − (%*Observed* for Clinician). State and transition frequency distributions that are statistically significantly different between patients and clinicians (*P* <0.05 by Mann Whitney U tests) are indicated by outlining states in black solid (*vs*. dashed) circles and drawing transition arrows with solid (*vs*. dashed) lines.

All eight states and eleven of the sixteen transitions differed significantly (*P* <0.05) between patients and clinicians (Fig 3; see S1 and S2 Tables for the corresponding *P* values). The difference CODYM between patients and clinicians helps one to visualize the overall dynamics of alternating patterns in information flow between patients and clinicians, where transitions in warm colors are more often taken by patients and transitions in cool colors are more often taken by clinicians. Most notably, it is evident that the LSL state is much more frequent prior to patient turns than clinician turns and is most often followed by a short patient turn, whereas the SLS state is much more frequent prior to clinician turns than patient turns and is most often followed by a long clinician turn. This implies that clinicians, rather than patients, are most often imparting more information in one-way information exchanges. It can also be seen that patients are more likely to initiate transition into the state SSS (short two-way information exchanges) whereas clinicians are more often the first to transition out of it, while the opposite is true of the state LLL (long two-way information exchanges).

### Temporal changes in normative patterns of information flow

**Fig 4:**
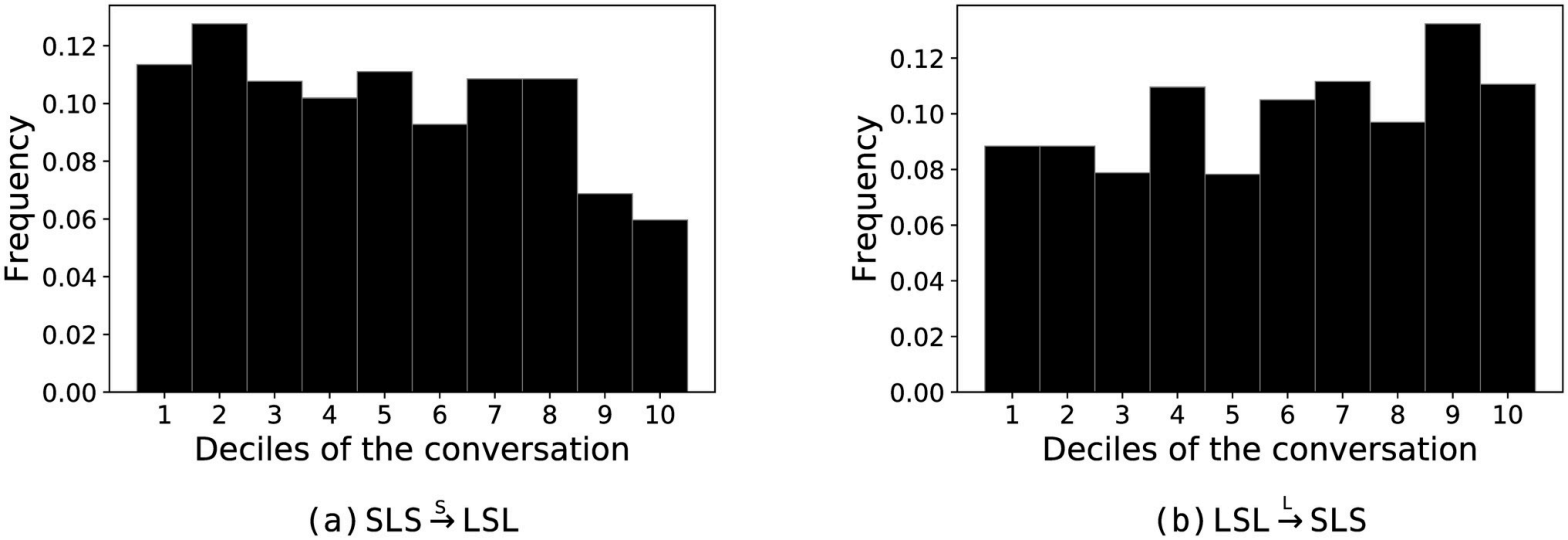
Temporal changes in 1-way information flow in the PCCRI corpus. Histograms of temporal changes in transition frequencies for patient turns in 3^rd^-order CODYMs for one-way information exchanges (a) from patient-to-clinician, and (b) from clinician-to-patient, over 10 conversational deciles that were subsequently averaged over all conversations in the PCCRI corpus and normalized, such that the sum of all bins is 1.0.

Some transitions show distinct patterns of change in frequency across temporal deciles of conversations (S6 and S7 Figs). Of particular interest is the observation that there is a general decrease in one-way information exchanges from patient-to-clinician over the course of the conversation (Fig 4a) with a corresponding increase in the one-way information flow from clinician-to-patient (Fig 4b).

### Contextualization by expression of distressing emotion

**Fig 5:**
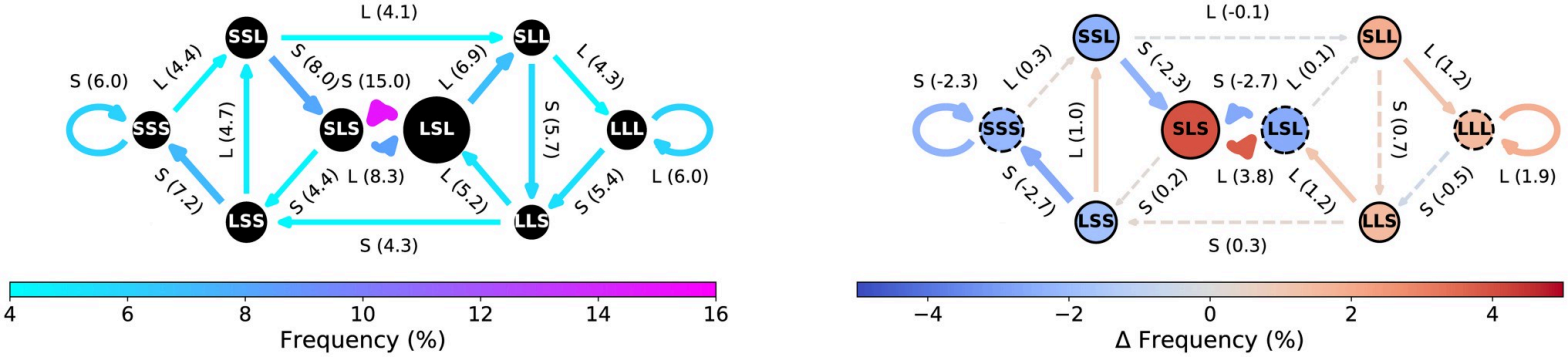
CODYMs by emotional content in PCCRI corpus. (Left) Normative patterns of patient turns for conversations where anger or fear are expressed. (Right) Difference CODYM, where Δ*Frequency* = (%*Observed* with anger or fear) − (%*Observed* without anger or fear). State and transition frequency distributions that are statistically significantly different (*P* <0.05 by Mann Whitney U tests) between patient turns in conversations with and without expressions of anger or fear are indicated in the difference CODYM by outlining states in black solid (*vs*. dashed) circles and drawing transition arrows with solid (*vs*. dashed) lines.

The expression of anger and fear occurs during L patient turns 68.8% and 64.3% of the time, respectively, whereas only 41% of all patient turns were L. The normative CODYM for patient turns in the 55 conversations where anger or fear are expressed by the patient is shown in Fig 5 (left) and the difference CODYM between the 55 conversations with *vs*. the 62 conversations without expressions of anger or fear is shown in Fig 5 (right); S3 and S4 Tables for the means and *P* values of all states and transitions. In the difference CODYM, transitions shown in warm colors occur more frequently in conversations where anger or fear are expressed, and those shown in cool colors occur more frequently in conversations without expressions of anger or fear. It is evident that conversations with these distressing emotions have significantly more one-way information flow from patients-to-clinicians and significantly less one-way information flow from clinicians-to-patients, than do conversations with no anger or fear expressed. In addition, in conversations that include patient expressions of anger or fear, we see a significant increase in patient transitions to long two-way information exchanges along with a significant decrease in patient transitions to short two-way information exchanges.

## Discussion

We have shown how CODYM analysis enables one to quantify, visualize, and compare high-level patterns in conversational dynamics from one or more conversations. We have made a deliberate choice to keep CODYMs simple, requiring only sequences of binarized turn lengths and using memory of only 3 turns. We have made these choices in order to facilitate interpretability, ensure adequate sample sizes for the corpus analyzed, and to protect privacy. However, as exemplified by our analyses of conversations that do or don’t contain audible expressions of anger or fear, even simple turn-length-based CODYMs can be contextualized to consider additional verbal or non-verbal features, if such are known.

CODYM visualizations effectively summarize the overall patterns of sequential dependencies in speaker turn lengths in concise plots, whose size and complexity are independent of the number of turns being analyzed. This facilitates rapid identification and comparison of patterns of information flow in sequences of turns that may include all or parts of one or many conversations. Since CODYM construction does not require access to detailed conversational content, the approach completely preserves the privacy of conversational participants and can conceptually be incrementally constructed in real-time, precluding the need for storage or transcription of conversations (as discussed further in Conclusions and Future Work). Although CODYMs can be contextualized and can be used to study temporally discretized patterns in information flow, they are not natively designed to capture the turn-level flow of concepts in individual conversations. CODYMs and Discursis [29] are thus complementary methods for visualizing information flow in conversations at different scales and levels of detail.

Conversation has generally been observed to be asymmetric, with a single dominant speaker holding the floor much of the time [58]. During these one-way information exchanges in dyadic conversations, the typical alternating pattern of speakers results in one speaker taking all the long turns (being the “talker”) while the other takes the short turns (being the “listener”). This is particularly true in institutional settings with defined speaker roles [58], so we expect it will be prevalent in serious illness conversations during which clinicians convey, and try to help the patient fully understand, the range of available treatment options. Consistent with this expectation, one-way information exchanges from clinician-to-patient were observed to be the most common information sharing pattern in the PCCRI corpus, occurring significantly more often than expected by chance (Fig 2). While patients adopt the talker role less often than clinicians do (Fig 3), they still do so more frequently than expected by chance in these serious illness conversations (Fig 2). In pooled patient turns, over all conversations in this study, one-way information exchanges from patient-to-clinician generally declined over the course of the conversations, with a marked drop near the end of the conversations (Fig 4a).

Patient expressions of anger and fear occur disproportionately often in long turns, and in conversations where fear or anger are expressed we observe a significant increase in the overall frequency of one-way patient-to-clinician information exchanges (Fig 5). Anger and fear manifest frequently in palliative care conversations [57], and have been found to have therapeutic effects. Being able to process and express distressing emotions is linked to improved health outcomes for patients with cancer [59], and it has recently been shown in the PCCRI corpus that expression of anger, in particular, is associated with improvement in how much patients feel heard and understood by the clinical team following a palliative care consult [53].

One possible explanation for the observed patterns in one-way information exchanges is that the palliative care clinicians in the PCCRI, trained to be highly skilled communicators, are taking on the role of “good listener” [58] during portions of the conversation in which difficult topics are being discussed, using short turns as a means of encouraging the patients to express their values and preferences relating to available treatment options, and providing an opportunity for them to express distressing emotions such as anger or fear. As the one-way patient-to-clinician information exchanges abate, these serious illness conversations may then naturally come to a close.

## Conclusions and future work

We have presented a novel approach to quantify and visualize overall patterns in the dynamics of information flow in conversations with CODYMs (COnversational DYnamics Models). CODYMs are the first Markov Model to use speaker turn length as the fundamental unit of information and the first model of any type to provide concise, high-level, quantitative summaries of overall dependencies in sequences of speaker turn lengths. This new approach facilitates identification and comparison of normative patterns of information flow across sequences of turns from one or more conversations, in context-independent or context-dependent ways. CODYMs complement existing qualitative and quantitative approaches for studying conversational dynamics, and thus provide a new tool for conversation analysis. We provide open source code for populating, visualizing, and contextualizing CODYMs [60].

To demonstrate the method, we applied CODYM analysis to a unique and important corpus of palliative care consultations with seriously ill patients. We discovered normative patterns of information flow in these conversations that differ between patients and clinicians, and between conversations with and without expressions of anger or fear. While these normative patterns are interesting in their own right, they may also have practical applications. For example, it would be interesting to compare normative CODYMs from in-person palliative care consultations to those conducted remotely, to see how telehealth platforms impact the patterns of information flow between patients and clinicians. Similarly, CODYM analysis of mock consultations between clinicians-in-training and actors portraying seriously ill patients could be used to assess how closely the information flow patterns of these training scenarios reflect those observed in palliative care consultations with real patients. In future work, we plan to expand our CODYM analysis of the PCCRI corpus in a variety of ways, including contextualization of turns surrounding “connectional silences” that have recently been identified in the PCCRI audio recordings [54] and are currently being annotated in the transcripts. If associations can be found between CODYM patterns and quality indicators of healthcare conversations (e.g., the degree to which patients feel heard and understood [61], a measure that is currently being considered for widespread use [62]), these could provide valuable insights for institutions seeking to improve the quality of conversations with seriously ill patients.

It is not clear whether the conversational “fingerprints” uncovered in the PCCRI corpus are unique to serious illness conversations, or represent more general conversational paradigms in healthcare (or other contexts). We suspect that the frequency of one-way patient-to-clinician information exchanges, and long two-way information exchanges, may be higher in serious illness communication relative to conversations in other clinical contexts (e.g., [63]). It will be fascinating to compare CODYMs across a wide variety of corpora from different languages, cultures, and contexts (including online conversations), to reveal which patterns of information flow in conversations are universal, and which are unique to certain settings.

Conversation analysis has traditionally been a discipline reliant on manual transcription of conversations with highly detailed annotations [6]. This is a resource-intensive process that requires full access to the often very private content of conversations. Indeed, the CODYM analyses presented here used transcriptions of audio-recordings of sensitive serious illness conversations. However, a time-based (rather than word count) definition of turn length would facilitate real-time automation and analysis of conversational dynamics, precluding the need for transcription or even storage of conversational audio, thus completely protecting privacy. Large numbers of conversations are already taking place in a medium that is natively capable of capturing conversational data appropriate for automated CODYM analysis. For example, many popular video conferencing services already incorporate tools that automate the detection of speaker turns, and such services have exploded in popularity in the wake of the Covid-19 global pandemic. Ongoing advances in the automated detection of conversational features including speaker recognition [64, 65], emotion [66, 67], conversational pauses [54], empathy [68, 69], gaze patterns [70], and word recognition [71], will facilitate real-time analysis and contextualization of CODYMs. Ultimately, we foresee a fully-automated pipeline for CODYM analyses, with no compromise to the privacy of conversational content.

As more conversational data become available, whether as transcriptions or through real-time processing, CODYMs have the potential to be a valuable tool for studying high level patterns of information flow in a wide variety of contexts and contributing to our understanding of how to have more effective conversations. Such a tool could be of practical utility in training and assessment of high quality communication in healthcare and other application domains, while also yielding new theoretical insights into conversational dynamics across languages, cultures, and contexts.

## Supporting information

S1 Dataset Minimal dataset for the PCCRI corpus. Spreadsheet containing anonymized data associated with the 117 PCCRI conversations analyzed here, sufficient to recreate the results presented in this paper. For each of the 27,597 turns, we include the conversation number, turn number, number of words, speaker (0 = patient, 1 = clinician, 2 = unknown), whether anger was audibly detected (0 = not detected, 1 = detected), and whether fear was audibly detected (0 = not detected, 1 = detected).
(CSV)

S1 Fig Words per turn in PCCRI corpus. The number of words per turn, for each of the 27,597 turns in the 117 PCCRI conversations analyzed.
(PDF)

S2 Fig Binarization of turn length in PCCRI corpus. Shannon entropy (information content) of transitions (red curve through circles, left *y*-axis) and the percentage of long turns for varying short/long thresholds (blue curve through squares, right *y*-axis) in a 3^rd^-order CODYM of the 117 PCCRI conversations analyzed. Shannon Entropy is calculated *S* = ∑_*i*_ *f*_*i*_ log *f*_*i*_ for the frequency *f*_*i*_ of each transition. The short/long threshold is defined such that for a threshold, *t*, any turn with *t* or more words is considered long. For all experiments in this study, we define short turns to be 7 or fewer words and long turns to be 8 or more words.
(PDF)

S3 Fig State distributions in PCCRI corpus. The distribution of each state in a 3^rd^-order CODYM, stratified by patient and clinician turns, across all 117 PCCRI conversations analyzed. Each distribution is labeled by patient (P) or clinician (C) turns, the state, and parenthetically the mean and median values, in that order.
(PDF)

S4 Fig Transition distributions of short turns in PCCRI corpus. The distribution of frequencies on each **short** transition in 3^rd^-order CODYMs, stratified by patient and clinician turns, across all 117 PCCRI conversations analyzed. Each distribution is labeled by patient (P) or clinician (C) turns, the transition, and parenthetically the mean and median values, in that order.
(PDF)

S5 Fig Transition distributions of long turns in PCCRI corpus. The distribution of frequencies on each **long** transition in 3^rd^-order CODYMs, stratified by patient and clinician turns, across all 117 PCCRI conversations analyzed. Each distribution is labeled by patient (P) or clinician (C) turns, the transition, and parenthetically the mean/median values.
(PDF)

S6 Fig Temporal changes in transitions of short turns in PCCRI corpus. Histograms of transition frequencies of all **short** turns in 3^rd^-order CODYMs over 10 conversational deciles (normalized, such that the sum of all bins is 1.0), stratified by the patient and clinician turns for the 117 PCCRI conversations analyzed.
(PDF)

S7 Fig Temporal changes in transitions of long turns in PCCRI corpus. Histograms of transition frequencies of all **long** turns in 3^rd^-order CODYMs over 10 conversational deciles (normalized, such that the sum of all bins is 1.0), stratified by the patient and clinician turns for the 117 PCCRI conversations analyzed.
(PDF)

S1 Table Significance tests of state distributions in PCCRI corpus. *P* values comparing the state distributions of 3^rd^-order CODYMs for the 117 PCCRI conversations analyzed, stratified by patient and clinician (shown in S3 Fig), of observed patient *vs*. observed clinician (using Mann Whitney U tests), and of observed patient *vs*. null patient models and observed clinician *vs*. null clinician models (by comparing to empirically derived probability distributions, as described in the text).
(PDF)

S2 Table Significance tests of transition distributions in PCCRI corpus. *P* values comparing the transition distributions of 3^rd^-order CODYMs for the 117 PCCRI conversations analyzed, stratified by patient and clinician (shown in S4 and S5 Figs), of observed patient *vs*. observed clinician (using Mann Whitney U tests), and of observed patient *vs*. null patient models and observed clinician *vs*. null clinician models (by comparing to empirically derived probability distributions, as described in the text).
(PDF)

S3 Table State comparisons with and without anger or fear. Mean values of state distributions of 3^rd^-order CODYMs for patient turns in the 117 PCCRI conversations analyzed, stratified by conversations with or without patient audible expressions of anger or fear. *P* values are from Mann Whitney U tests used to compare the underlying distributions of corresponding states.
(PDF)

S4 Table Transition comparisons with and without anger or fear. Mean values of transition distributions of 3^rd^-order CODYMs for patient turns in the 117 PCCRI conversations analyzed, stratified by conversations with or without patient audible expressions of anger or fear. *P* values are from Mann Whitney U tests used to compare the underlying distributions of corresponding transitions.
(PDF)
