# Supplementary figures and images for: A general model of conversational dynamics and an example application in serious illness communication
Source: PLoS One. 2021 Jul 1;16(7):e0253124. doi: 10.1371/journal.pone.0253124 (PMC8248661; doi:10.1371/journal.pone.0253124)

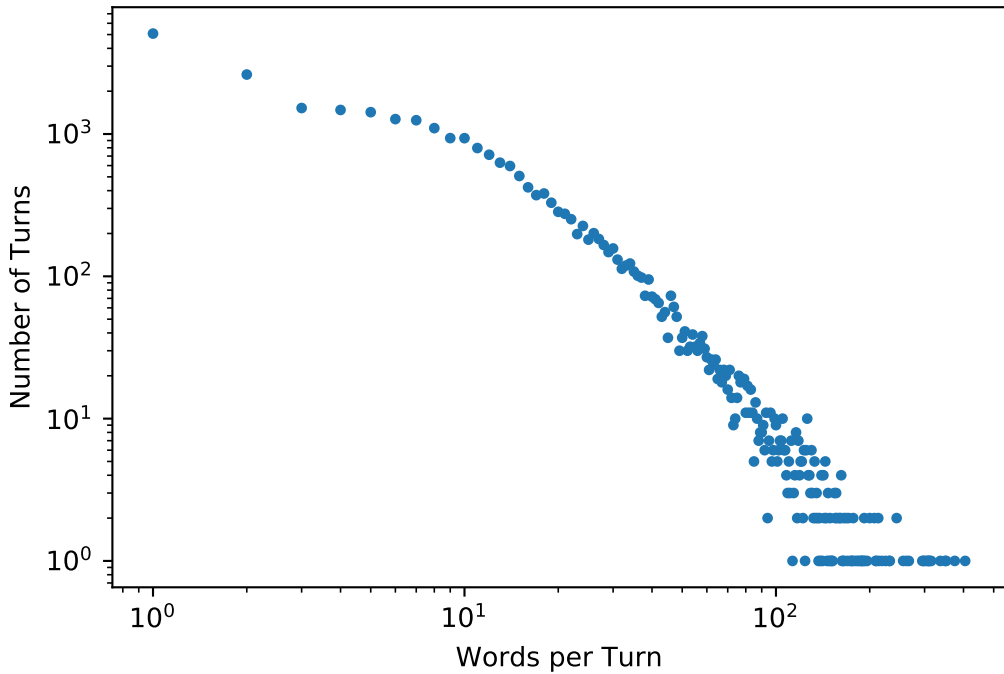

Supplement: S1 Fig — The number of words per turn, for each of the 27,597 turns in the 117 PCCRI conversations analyzed. (PDF) [file pone.0253124.s002.pdf]

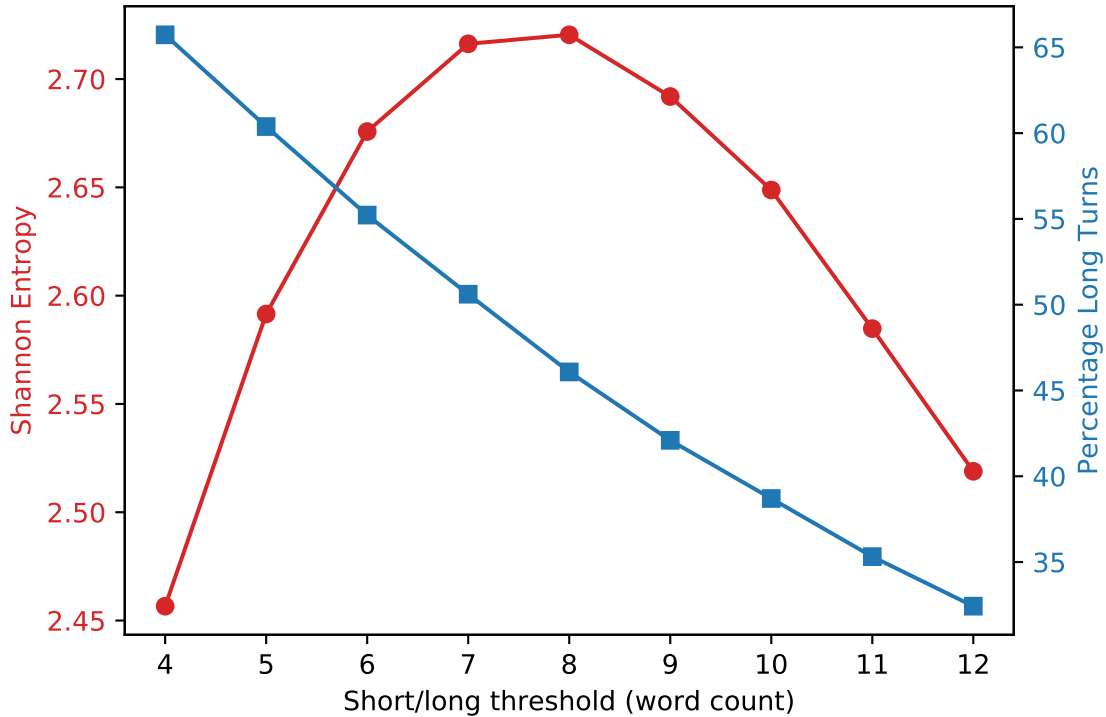

Supplement: S2 Fig — Shannon entropy (information content) of transitions (red curve through circles, left y-axis) and the percentage of long turns for varying short/long thresholds (blue curve through squares, right y-axis) in a 3rd-order CODYM of the 117 PCCRI conversations analyzed. Shannon Entropy is calculated S = ∑i fi log fi for the frequency fi of each transition. The short/long threshold is defined such that for a threshold, t, any turn with t or more words is considered long. For all experiments in this study, we define short turns to be 7 or fewer words and long turns to be 8 or more words. (PDF) [file pone.0253124.s003.pdf]

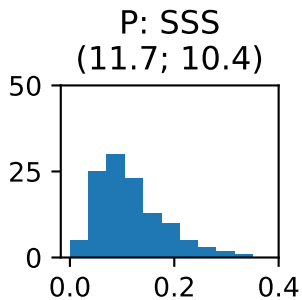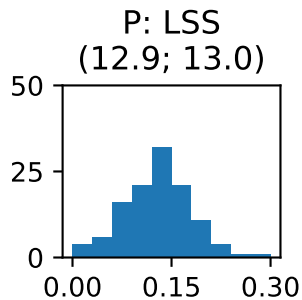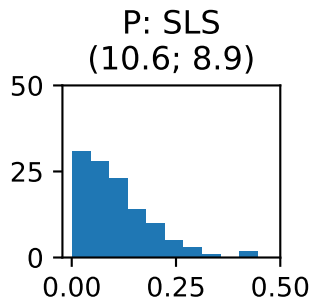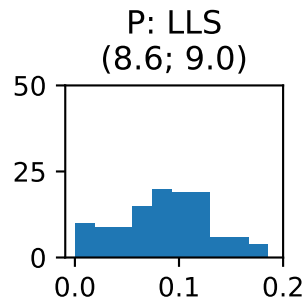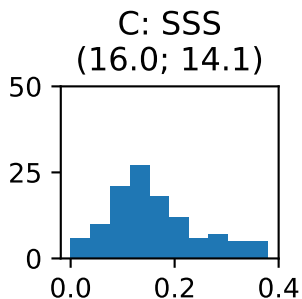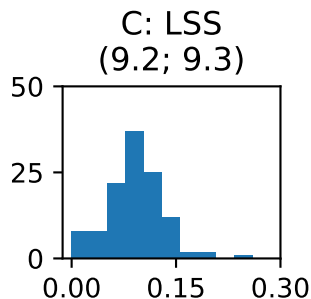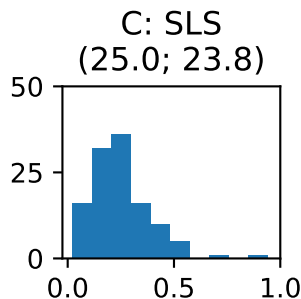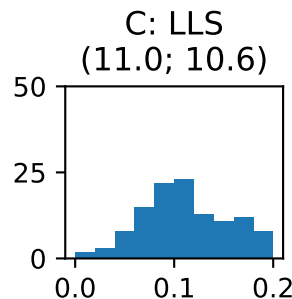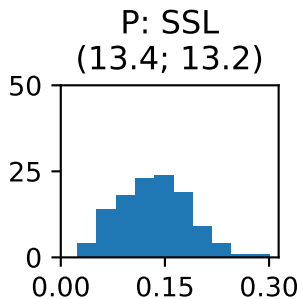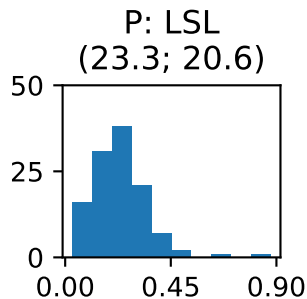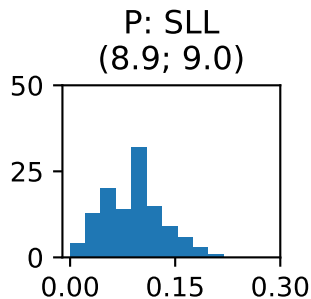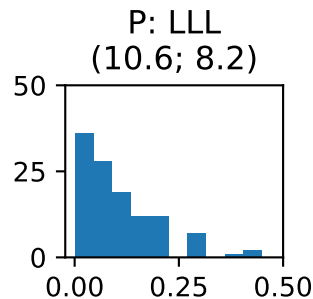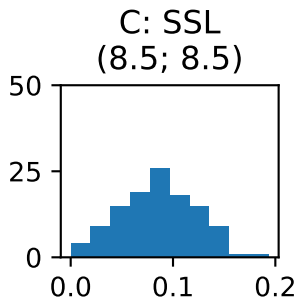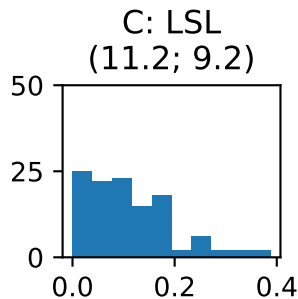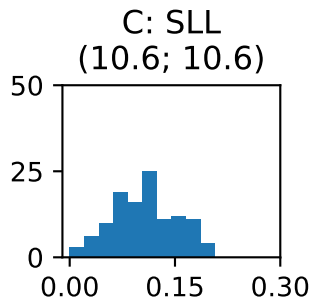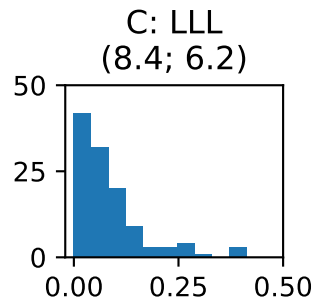

Supplement: S3 Fig — The distribution of each state in a 3rd-order CODYM, stratified by patient and clinician turns, across all 117 PCCRI conversations analyzed. Each distribution is labeled by patient (P) or clinician (C) turns, the state, and parenthetically the mean and median values, in that order. (PDF) [file pone.0253124.s004.pdf]
