# Supplementary material for: A general model of conversational dynamics and an example application in serious illness communication
Source: PLoS One. 2021 Jul 1;16(7):e0253124. doi: 10.1371/journal.pone.0253124 (PMC8248661; doi:10.1371/journal.pone.0253124)
Supplement: S5 Fig — The distribution of frequencies on each long transition in 3rd-order CODYMs, stratified by patient and clinician turns, across all 117 PCCRI conversations analyzed. Each distribution is labeled by patient (P) or clinician (C) turns, the transition, and parenthetically the mean/median values. (PDF) [file pone.0253124.s006.pdf]

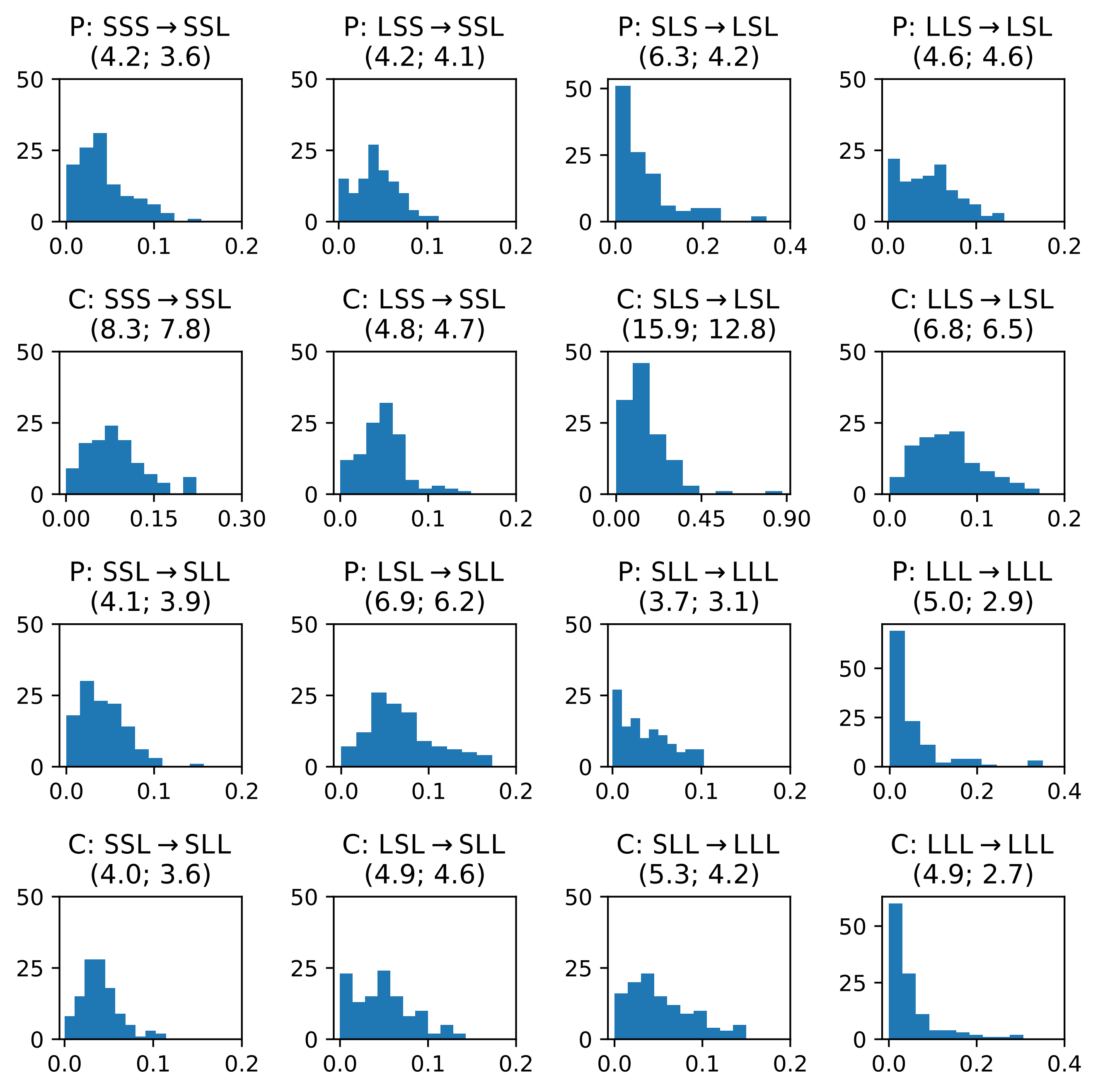

P: SSS → SSL
(4.2; 3.6)
P: LSS → SSL
(4.2; 4.1)
P: SLS → LSL
(6.3; 4.2)
P: LLS → LSL
(4.6; 4.6)
C: SSS → SSL
(8.3; 7.8)
C: LSS → SSL
(4.8; 4.7)
C: SLS → LSL
(15.9; 12.8)
C: LLS → LSL
(6.8; 6.5)
P: SSL → SLL
(4.1; 3.9)
P: LSL → SLL
(6.9; 6.2)
P: SLL → LLL
(3.7; 3.1)
P: LLL → LLL
(5.0; 2.9)
C: SSL → SLL
(4.0; 3.6)
C: LSL → SLL
(4.9; 4.6)
C: SLL → LLL
(5.3; 4.2)
C: LLL → LLL
(4.9; 2.7)
