# Supplementary material for: A general model of conversational dynamics and an example application in serious illness communication
Source: PLoS One. 2021 Jul 1;16(7):e0253124. doi: 10.1371/journal.pone.0253124 (PMC8248661; doi:10.1371/journal.pone.0253124)
Supplement: S6 Fig — Histograms of transition frequencies of all short turns in 3rd-order CODYMs over 10 conversational deciles (normalized, such that the sum of all bins is 1.0), stratified by the patient and clinician turns for the 117 PCCRI conversations analyzed. (PDF) [file pone.0253124.s007.pdf]

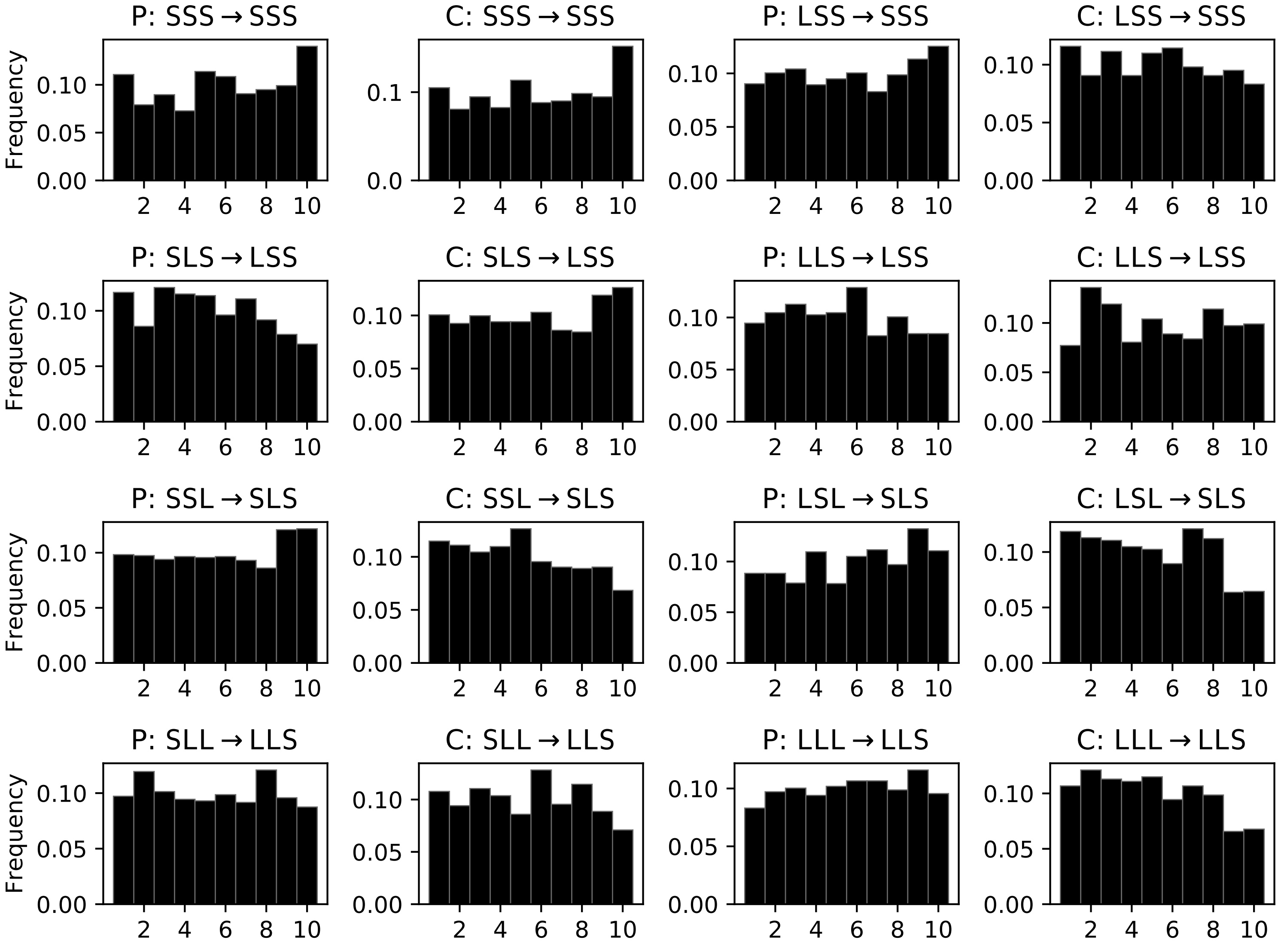

P: SSS → SSS
C: SSS → SSS
P: LSS → SSS
C: LSS → SSS
P: SLS → LSS
C: SLS → LSS
P: LLS → LSS
C: LLS → LSS
P: SSL → SLS
C: SSL → SLS
P: LSL → SLS
C: LSL → SLS
P: SLL → LLS
C: SLL → LLS
P: LLL → LLS
C: LLL → LLS
Frequency
0.00
0.05
0.10
0.0
0.1
2
4
6
8
10
