# Supplementary material for: A general model of conversational dynamics and an example application in serious illness communication
Source: PLoS One. 2021 Jul 1;16(7):e0253124. doi: 10.1371/journal.pone.0253124 (PMC8248661; doi:10.1371/journal.pone.0253124)
Supplement: S7 Fig — Histograms of transition frequencies of all long turns in 3rd-order CODYMs over 10 conversational deciles (normalized, such that the sum of all bins is 1.0), stratified by the patient and clinician turns for the 117 PCCRI conversations analyzed. (PDF) [file pone.0253124.s008.pdf]

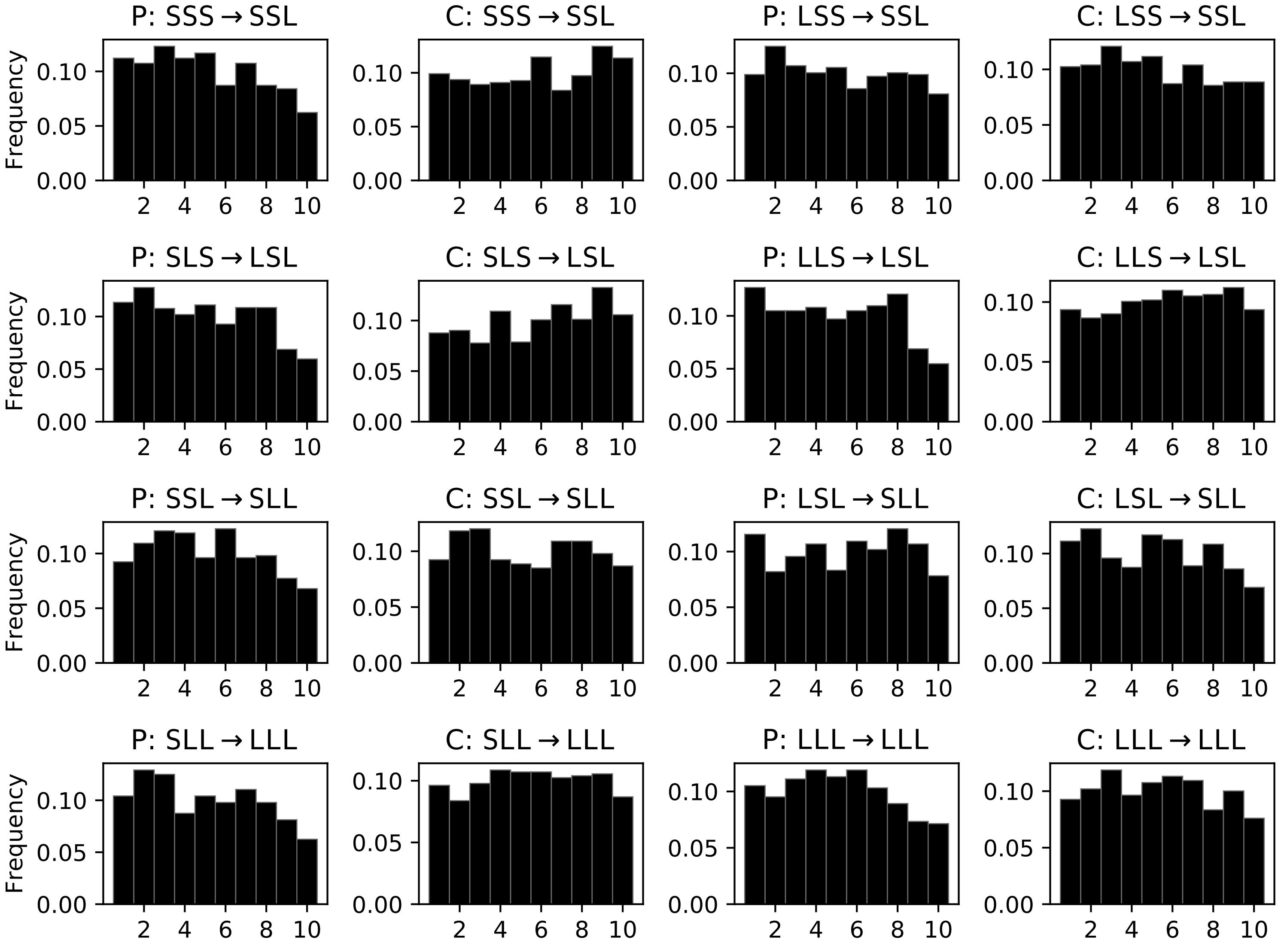

P: SSS → SSL
C: SSS → SSL
P: LSS → SSL
C: LSS → SSL
P: SLS → LSL
C: SLS → LSL
P: LLS → LSL
C: LLS → LSL
P: SSL → SLL
C: SSL → SLL
P: LSL → SLL
C: LSL → SLL
P: SLL → LLL
C: SLL → LLL
P: LLL → LLL
C: LLL → LLL
Frequency
0.00
0.05
0.10
2
4
6
8
10
