# Supplementary material for: A general model of conversational dynamics and an example application in serious illness communication
Source: PLoS One. 2021 Jul 1;16(7):e0253124. doi: 10.1371/journal.pone.0253124 (PMC8248661; doi:10.1371/journal.pone.0253124)
Supplement: S1 Table — P values comparing the state distributions of 3rd-order CODYMs for the 117 PCCRI conversations analyzed, stratified by patient and clinician (shown in S3 Fig), of observed patient vs. observed clinician (using Mann Whitney U tests), and of observed patient vs. null patient models and observed clinician vs. null clinician models (by comparing to empirically derived probability distributions, as described in the text). (PDF) [file pone.0253124.s009.pdf]

S1 Table. Significance tests of state distributions in PCCRI corpus. P values comparing the state distributions of 3rd-order CODYMs for the 117 PCCRI conversations analyzed, stratified by patient and clinician (shown in S3 Fig), of observed patient *vs.* observed clinician (using Mann Whitney U tests), and of observed patient *vs.* null patient models and observed clinician *vs.* null clinician models (by comparing to empirically derived probability distributions, as described in the text).

State	Patient *vs.* Clinician	Patient *vs.* Null	Clinician *vs.* Null
SSS	< 0.001	< 0.001	< 0.001
LSS	< 0.001	< 0.001	< 0.001
SLS	< 0.001	< 0.001	< 0.001
LLS	< 0.001	0.006	0.010
SSL	< 0.001	0.002	< 0.001
LSL	< 0.001	< 0.001	< 0.001
SLL	0.002	0.009	< 0.001
LLL	0.014	< 0.001	< 0.001
