# Supplementary material for: A general model of conversational dynamics and an example application in serious illness communication
Source: PLoS One. 2021 Jul 1;16(7):e0253124. doi: 10.1371/journal.pone.0253124 (PMC8248661; doi:10.1371/journal.pone.0253124)
Supplement: S2 Table — P values comparing the transition distributions of 3rd-order CODYMs for the 117 PCCRI conversations analyzed, stratified by patient and clinician (shown in S4 and S5 Figs), of observed patient vs. observed clinician (using Mann Whitney U tests), and of observed patient vs. null patient models and observed clinician vs. null clinician models (by comparing to empirically derived probability distributions, as described in the text). (PDF) [file pone.0253124.s010.pdf]

S2 Table. Significance tests of transition distributions in PCCRI corpus. P values comparing the transition distributions of 3rd-order CODYMs for the 117 PCCRI conversations analyzed, stratified by patient and clinician (shown in S4 Fig and S5 Fig), of observed patient *vs.* observed clinician (using Mann Whitney U tests), and of observed patient *vs.* null patient models and observed clinician *vs.* null clinician models (by comparing to empirically derived probability distributions, as described in the text).

Transition	Patient *vs.* Clinician	Patient *vs.* Null	Clinician *vs.* Null
SSS$\xrightarrow{S}$SSS	0.234	< 0.001	0.199
SSS$\xrightarrow{L}$SSL	< 0.001	0.002	< 0.001
LSS$\xrightarrow{S}$SSS	< 0.001	0.017	< 0.001
LSS$\xrightarrow{L}$SSL	0.038	< 0.001	0.010
SLS$\xrightarrow{S}$LSS	< 0.001	< 0.001	0.241
SLS$\xrightarrow{L}$LSL	< 0.001	< 0.001	< 0.001
LLS$\xrightarrow{S}$LSS	0.171	< 0.001	< 0.001
LLS$\xrightarrow{L}$LSL	< 0.001	0.003	0.084
SSL$\xrightarrow{S}$SLS	< 0.001	0.404	0.023
SSL$\xrightarrow{L}$SLL	0.341	< 0.001	< 0.001
LSL$\xrightarrow{S}$SLS	< 0.001	< 0.001	< 0.001
LSL$\xrightarrow{L}$SLL	< 0.001	0.005	0.010
SLL$\xrightarrow{S}$LLS	0.474	0.428	0.133
SLL$\xrightarrow{L}$LLL	< 0.001	0.001	< 0.001
LLL$\xrightarrow{S}$LLS	< 0.001	< 0.001	< 0.001
LLL$\xrightarrow{L}$LLL	0.464	0.173	0.011
