# Supplementary material for: A general model of conversational dynamics and an example application in serious illness communication
Source: PLoS One. 2021 Jul 1;16(7):e0253124. doi: 10.1371/journal.pone.0253124 (PMC8248661; doi:10.1371/journal.pone.0253124)
Supplement: S3 Table — Mean values of state distributions of 3rd-order CODYMs for patient turns in the 117 PCCRI conversations analyzed, stratified by conversations with or without patient audible expressions of anger or fear. P values are from Mann Whitney U tests used to compare the underlying distributions of corresponding states. (PDF) [file pone.0253124.s011.pdf]

S3 Table. State comparisons with and without anger or fear. Mean values of state distributions of 3rd-order CODYMs for patient turns in the 117 PCCRI conversations analyzed, stratified by conversations with or without patient audible expressions of anger or fear. *P* values are from Mann Whitney U tests used to compare the underlying distributions of corresponding states.

State	With Emotion	Without Emotion	*P* value
SSS	10.5	12.7	0.0861
LSS	11.9	13.7	0.0097
SLS	12.7	8.8	0.0261
LLS	9.5	7.9	0.0343
SSL	12.2	14.5	0.0065
LSL	21.9	24.5	0.1485
SLL	9.9	8.0	0.0049
LLL	11.4	9.9	0.1549
