# Supplementary material for: A general model of conversational dynamics and an example application in serious illness communication
Source: PLoS One. 2021 Jul 1;16(7):e0253124. doi: 10.1371/journal.pone.0253124 (PMC8248661; doi:10.1371/journal.pone.0253124)
Supplement: S4 Table — Mean values of transition distributions of 3rd-order CODYMs for patient turns in the 117 PCCRI conversations analyzed, stratified by conversations with or without patient audible expressions of anger or fear. P values are from Mann Whitney U tests used to compare the underlying distributions of corresponding transitions. (PDF) [file pone.0253124.s012.pdf]

S4 Table. Transition comparisons with and without anger or fear. Mean values of transition distributions of 3rd-order CODYMs for patient turns in the 117 PCCRI conversations analyzed, stratified by conversations with or without patient audible expressions of anger or fear. *P* values are from Mann Whitney U tests used to compare the underlying distributions of corresponding transitions.

Transition	With Emotion	Without Emotion	*P* value
SSS $\xrightarrow{S}$ SSS	6.0	8.4	0.02
SSS $\xrightarrow{L}$ SSL	4.4	4.1	0.2109
LSS $\xrightarrow{S}$ SSS	7.2	9.8	0.0013
LSS $\xrightarrow{L}$ SSL	4.7	3.7	0.0247
SLS $\xrightarrow{S}$ LSS	4.4	4.3	0.3705
SLS $\xrightarrow{L}$ LSL	8.3	4.6	0.0017
LLS $\xrightarrow{S}$ LSS	4.3	4.0	0.1174
LLS $\xrightarrow{L}$ LSL	5.2	4.1	0.0206
SSL $\xrightarrow{S}$ SLS	8.0	10.3	0.0101
SSL $\xrightarrow{L}$ SLL	4.1	4.2	0.4815
LSL $\xrightarrow{S}$ SLS	15.0	17.7	0.1595
LSL $\xrightarrow{L}$ SLL	6.9	6.9	0.4414
SLL $\xrightarrow{S}$ LLS	5.7	5.0	0.079
SLL $\xrightarrow{L}$ LLL	4.3	3.2	0.0167
LLL $\xrightarrow{S}$ LLS	5.4	5.8	0.4082
LLL $\xrightarrow{L}$ LLL	6.0	4.1	0.015
